# Review and Assessment of Material, Method, and Predictive Modeling for Fiber-Reinforced Polymer (FRP) Partially Confined Concrete Columns

**DOI:** 10.3390/polym16101367

**Published:** 2024-05-10

**Authors:** Muhammad Usman Ghani, Nauman Ahmad, Kahsay Gebresilassie Abraha, Rana Zafar Abbas Manj, Muhammad Haroon Sharif, Li Wei

**Affiliations:** 1 Key Lab of Textile Science & Technology, Ministry of Education, College of Textiles, Donghua University, Shanghai 201620, China; 420011@mail.dhu.edu.cn (M.U.G.); kahsaymam6@gmail.com (K.G.A.); 2 Department of Civil Engineering, Tongji University, Shanghai 200092, China; naumanmeo135@gmail.com; 3 Department of Textile Engineering, Aksum University, Aksum P.O. Box 1010, Tigrai, Ethiopia; 4 State Key Laboratory for Modification of Chemical Fibers and Polymer Materials, College of Materials Science and Engineering, Donghua University, Shanghai 201620, China; ranazafar@mail.dhu.edu.cn (R.Z.A.M.); bhatiharoon@gmail.com (M.H.S.); 5 Center for Civil Aviation Composites, Donghua University, Shanghai 201620, China

**Keywords:** fiber-reinforced polymer (FRP), partial confinement, predictive modeling, finite element modeling (FEM), stress–strain behavior

## Abstract

The repairing and strengthening of concrete structures using external and internal partial confinements are inevitable in the construction industry due to the new standards and rapid developments. The conventional materials and methods of confinement are unable to meet modern safety and functional standards. The fiber-reinforced polymer (FRP) enhances the strength and ductility of deteriorating and new concrete columns by reducing lateral confinement pressure and resistance against seismic shocks. The precise methods of partial confinement are inevitable for effective FRP-concrete bonding, durability, and cost-effectiveness under different loading conditions and to cope with external environmental factors. Predictive modeling and simulation techniques are pivotal for the optimization of confinement materials and methods by investigating the FRP-concrete novel confinement configurations, stress–strain responses, and failure modes. The novel materials and methods for concrete columns’ partial confinement lack high compressive strength, ductility, chemical attack resistivity, and different fiber orientation impacts. This review provides an overview of recent confinement materials, novel methods, and advanced modeling and simulation techniques with a critical analysis of the research gaps for partial FRP confinement of concrete columns. The current challenges and future prospects are also presented.

## 1. Introduction

Civil structures such as bridges and mining sites are prone to deterioration, and few due to natural disasters such as earthquake damage also require rapid repair. The lack of design capacity of traditional repairment methods [1], along with other drawbacks like heavy weight, corrosiveness, and high price, has urged to the development of new materials and precise methods. The unique features and properties of advanced materials are high tensile strength, lightweight, durability, strong bonding, elasticity against seismic forces, and corrosion resistance [2,3,4]. Therefore, excellent material properties and precise methods are necessary to retrofit the existing concrete structures and enhance their durability, safety, and performance.

The research of composite materials as a substitute for traditional materials has seen rapid growth in the manufacturing industry [2]. The applications of glass and carbon fiber reinforced polymer (GFRP and CFRP) composites are numerous, ranging from airplane to cell phone manufacturing [5,6]. The fiber reinforce polymer (FRP) composites are also becoming common to improve the compressive strength of existing columns of concrete due to the high ratio of strength to weight along with resistance to corrosion [7,8,9]. To further optimize the performance of FRP, reduce cost, enhance environment friendliness, and tailor the material to specialized applications, further research is necessary.

Along with the materials, the methods of confinement are equally important for the effective strengthening of concrete columns. FRP external reinforcements (strips) are usually suitable for repair, while internal reinforcements (hoop ties and spirals) are used for new structures. The external wrappings are further classified into full and partial confinement. After extensive research on full confinement, the current focus is on partial confinement due to design flexibility, ductility, and cost-effectiveness [10]. For a certain amount of axial deformation capacity in concrete columns, the partial wrappings of FRP are suggested [11]. As compared to full wraps, partial wrapping will result in fewer materials and easier and faster wrappings. A study proved that two layers of CFRP partial confinement give better material performance and are economical as compared to full confinement [12]. On the other hand, less material means less waste generation during installation and dismantling, which leads to lower carbon emissions. Due to limited work on partial confinement, the compressive behavior after partial confinement of FRP on the concrete columns for large-scale civil structures is still unclear. Therefore, design-oriented models and simulations are necessary to study the influence of key factors on partial confinement performance.

Extensive research has been performed on accurate and precise analysis/design-oriented stress–strain models based on the dilation behavior of fully FRP-confined RC/concrete columns [13,14,15,16,17]. However, the various stress–strain analytical models based on limited data or parametric values have shown poor performance when adapted for large-scale projects [18]. Therefore, for partially confined concrete columns, it is imperative to develop more precise and efficient models under compressive loading for safety against failure modes, optimal ratio, and the width and spacing of wraps [19]. These models on test specimens would be effective for the mechanical properties of deteriorating large-scale civil structures to develop large-scale models for the partial confinement of the deteriorating parts.

Previously, there had not been a comprehensive study on partial confinement materials and methods optimization through predictive modeling approaches. This review paper comprises three major sections: confinement materials, partial confinement methods, and modeling approaches for partial FRP confinement. Section 1 comprises the introduction part of the study. Section 2 explains the confinement materials for concrete columns, focusing on the design properties of wrapping materials and achieving performance after concrete columns confinement. Section 3 analyzes the advanced methods for the effective partial confinement of concrete columns, describing recommended partial confinement configurations. Section 4 illustrates the predictive modeling and simulation approaches for the partial confinement of concrete columns by FRP wrappings. Section 5 is the comparative analysis between confinement materials, methods, and modeling approaches, highlighting the overall challenges and future perspectives.

## 2. Materials for FRP Wrapping and Specimen Strengthening

**Figure 1:**
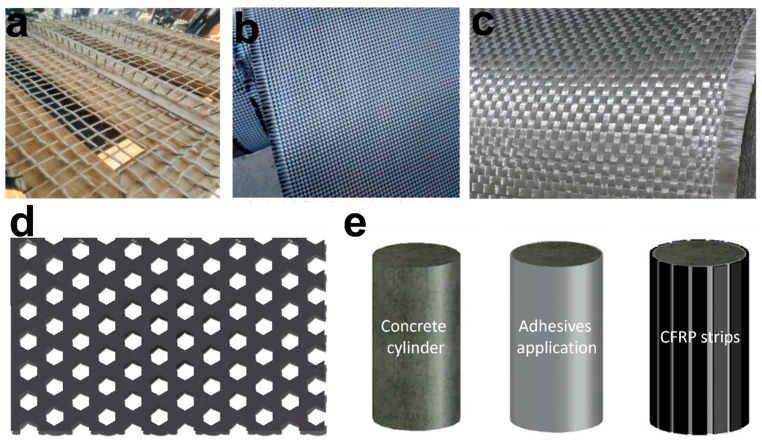
Fiber-reinforced polymer composites for the effective confinement of concrete columns. (**a**) The banana geotextile composed of geopolymer, banana fiber, and fly ash with various grid spacings for concrete confinement [20]. (**b**) A composite of fabric and epoxy resin, as a sheet of hybrid fabric reinforced polymer with glass and carbon to enhance the seismic performance of RC [21]. (**c**) The sheet of woven roving laminating material for reinforcement to provide increased strength and quick construction. (**d**) Structure of the triaxial woven fabric for concrete columns partial confinement and composed of carbon and glass, having different weft and warp configurations [19]. (**e**) Representing the fabrication process of CFRP strips in a longitudinal direction [22]. Source: (**a**,**c**,**d**) are from an open-access article distributed under the Creative Commons Attribution License. (**b**,**e**) reproduced with permission [21,22] Copyright © 2023, Elsevier.

Various efforts have been made to improve the comprehensive strength of unconfined concrete columns using traditional materials like steel. However, due to new composites research with improved properties, it is imperative to adapt them for the confinement of concrete structures. Therefore, the recent applications of composite materials in concrete confinement are mentioned hereby (Figure 1).

### 2.1. Carbon-FRP

A comprehensive experimental study was conducted on plain and reinforced confined concrete (FRP) only for circular specimens [23]. It consists of 60 CFRP-confined RC and 63 CFRP-confined plain specimens with an alteration in material properties and geometries. Carbon fiber (CF) sheets of the types M1, M2, and M3 were used. Among those, M3 had the highest rupture strain (2.00%) and tensile strength (4800 MPa). By using that designed methodology, there was a significant influence on confinement efficiency by the increment in compressive strength of confined concrete specimens. A stress–strain model was also developed for ultimate design conditions. This study also led to the prediction of hoop strain (εju) and partial factor (γj) for CFRPs. In general, it was suggested that the constant design factor (K1) had to be reflected critically. Further studies are needed for substandard and low-strength concrete. To increase the strength of hybrid fiber reinforced concrete (HFRC) specimens or cylinders (18 in number) confided with carbon fiber-reinforced polymer (CFRP) by checking their compressive stress–strain behavior axial compressive strain axial compressive strength for low strength HFRC specimens wrapped with CFRP sheets [24]. The HFRC is composed of polypropylene fibers and steel fibers. Two types of HFRC with a low strength of 12.5 MPa and 16.5 MPa were fabricated for the experiment. The results showed an increase in CS for the 12.5 MPa group for the single CFRP layer (115.7%), and for the double layer, it was increased to 130.7%. In the same manner, the CS for the 16.5 MPa group was increased for the single CFRP layer to 37.4% and for the double layer of CFRP to 112.6%. It is concluded that in terms of axial compressive strain and axial compressive strength, the CFRP confinement is more effective for low-strength HFRP as compared to high-strength HFRC. Ductility was also improved besides strength. It is recommended that in low-strength HFRC, the most effective factor for the enhancement of ductility is CFRP confinement. In another study, 27 non-confined and confined cylinders with spirals of a novel hybrid fiber-reinforced polymer (HFRP) were subjected to axial compressive loading [25]. Spiral types and spiral spacing were two variables in the study. The results indicated that the basalt fiber-reinforced polymer spiral and HFRP spiral confined specimens illustrated similar stress–strain relationships and compressive failure modes. An advantage of composite HFRP over CFRP spiral was that it showed larger actual fracture strain. HFRP spiral’s maximum strain reached 70%. However, for BFRP spirals, its value reached 50%. It was concluded that the ultimate stress and strain values for FRP spirally confined specimens were overestimated compared with the ultimate stress and strain for the FRP spiral-confined cylinders; the majority of the existing models overestimated their ultimate stress and strain. A model based on the strain compatibility principle and Poisson’s ratio was recommended to serve the purpose.

### 2.2. Glass-FRP

To further check the effectiveness of fiberglass, an experimental study was conducted on rectangular columns with a size of 150 mm × 300 mm casted under M20 and M40 grades, which were wrapped with GFRP sheets having thicknesses of 3 mm and 5 mm [26]. As the glass fiber had high elasticity modulus and tensile strength, the glass fiber-reinforced polymer was used in this study as the wrapping material. The results indicated that in the M20 grade, the specimen wrapped with 5 mm increased 5.182% more as compared to the specimens wrapped with 3 mm. Meanwhile, in M40, the specimen wrapped 5 mm increases to 2.47% more than the other specimens. The maximum strength was obtained by 5 mm wrapping. It was suggested that the thickness be increased, or an appropriate quantity of material be added for GFRP materials to resist creep. Ductile detailing could be best for GFRP concrete structural elements as compared to impact loading. The elastic modulus of GFRP is less as compared to steel and concrete, but it could be improved with more research. It is recommended that the GFRP design be improved to adapt better to various environmental changes, like high temperatures. The concrete columns could also be strengthened with a hybrid system comprising transverse GFRP wrapping and longitudinal CFRP laminates. Therefore, ref. [22] conducted a study on eccentrically loaded columns and developed a numerical and analytical model. To attain full capacity, the failure mode of CFRP wrapping the rebounding or buckling was altered to crushing by using GFRP wraps over longitudinal CFRPs. The strengthening and effectiveness of small-scale concrete columns under axial loading were demonstrated by the use of wrapping without longitudinal CFRP laminates rather than the proposed hybrid system. Axial capacity, lateral displacement at peak load and flexural capacity were added by 52%, 94%, and 105%, respectively, from enhancing the wrapping system by hybrid system. At the same time, the best performance level could be obtained by changing the load-deflecting curve for the slender specimens. It is recommended that further studies are needed to check the performance of this system by conducting cyclic loading and also considering eccentricities for earthquake-resistant structures.

The interaction between internal transverse reinforcement and FRCM jackets occurred in an experimental study [27]. The eight reinforced concrete (RC) columns were tested under axial load: four of them were bare specimens, while the remaining four were confined with an E-glass fiber cementitious matrix (GFRCM). The experimental variables were the presence of an FRCM jacket or not, two stirrups spacing values, and the geometry of the column cross-section (square or circular). The results demonstrated that the axial strain capacity and confined compressive strength were increased with the use of GFRCM jackets. The GFRCM jackets had a fiber exploitation ratio in the range of 17–40%, which was higher than that of CFRCM jackets, which was in the range of 3–23%. Further studies are needed to define the interaction between internal transverse reinforcement and FRCM jacket. Further, to minimize the impact of glass on the environment, a study [28] investigated numerical and experimental modeling on concrete cylinders of 28 days filled with colored waste glass (WG), which were confided with polypropylene textile to test their uni-axial compressive behavior. Mass fractions ranging from 0 to 15 percent with an increment step of 15% were used to replace sand and cement. In that experimental study, three types of confinement were used, including partial confinement concrete circular (PCCC), total confinement (TCC) and partial confinement concrete helical (PCCH). The highest resistant level of 34.78 MPa was shown by the specimen having 5% glass sand and 10% glass powder. When the CG content was enhanced to 15%, the strength of the concrete was decreased to 10%. The polypropylene textile configuration as an envelope enhanced the confinement efficiency of concrete made of recycled glass. When the three confinements were compared, the full confined illustrated the highest strength among others. It is recommended that by the bonding of composite materials, the mechanical properties could be enhanced. The FRP-concrete bonding could be enhanced using epoxy resin [29], primer, and surface preparation [30]. It is equally important to assess the bonding strength right after retrofitting and later passing designed years, especially under ambient conditions. Various tests are presented for this purpose, including the pull-out test [31]; the five major classes of test are classified as single shear type, double shear type, bending type, mixed-mode loading test, and direct tension type [32]. Nowadays, machine learning (ML) models are also being used to inculcate the increasing parameters and materials for better generalization, such as Gaussian Process Regression (GPR) and Adaptive Neuro-Fuzzy Interface system (ANFIS) [33]. Further, to manufacture new concrete, the promising alternative is the use of waste glass.

### 2.3. Hybrid Composites

To check optimal seismic performance, 10 reinforced concrete beams, and column joints were applied to the axial load with reversed cyclic loading [21]. The loadings simulated an earthquake and were tested under deflection, which was monitored. Fiber-reinforced polymer (FRP) was used to strengthen the joints, while the FRP system was composed of fiberglass, carbon fabric, and hybrid-braided FRP fabric. The damaged samples were repaired with FRP during the experiment and again retested. Aramis digital video camera was used to monitor cracking and strain fields. The outcome established that GFRP was more efficient than CFRP in upgrading deficient beam-column joints. To improve the dissipation energy and ductility of RC joints, the use of a hybrid sheet (carbon and glass) was recommended at a highly competitive cost. That study confirmed that in earthquake-prone areas, fiberglass had tremendous potential to enhance the ductility behavior of beam-column joints. To further check the effectiveness of carbon wrapping against seismic shocks, the 18 RC columns under quasi-static cyclic loading were investigated for seismic performance [34], and for the strengthening of the specimen’s steel, collar applications and CFRP wrapping were used. Two parameters that affected the seismic behavior of specimens were also considered: axial load ratio and concrete strength. The average ductility of the columns was increased using collar strengthening and CFRP wrapping by 61.7–100.8% and 38.8–108.6%, respectively, as compared to the reference columns. In the same scenario, the energy dissipation was also increased for collared columns and CFRP-wrapped columns by 379.3% and 347.2%, respectively. On the basis of the experimental observation, the thickness of a steel collar of less than 10 mm is recommended.

A novel technique was practiced in a research study to combine the engineered cementitious composite (ECC) with basalt textile as an effective material for the confinement of concrete columns [35]. To create pure hoop tensile stress on the confinement layers, an axial load was applied at the concrete core. The results indicated that ECC could be effectively bounded with textile fiber, and it is an alternative that overcame the traditional strengthening drawbacks. A new confinement model was also created for this novel technique’s compressive strength prediction. There was a considerable improvement in the ductility and load-carrying capacity when BFTR ECC or ECC were used for the confinement of circular columns. It is suggested to find some new fiber grid that could be used with ECC as a combination. In another study, the concrete columns were wrapped with polyethylene naphthalate fiber-reinforced polymer (PEN FRP), and the effects of confinement on concrete specimens under an axial compression load were investigated [36]. Then, a comparison was conducted between them and those who were wrapped with basalt FRP (BFRP). The test variables were a number of FRP layers and two various kinds of FRP confinement. The stress–strain relationship indicated that PEN FRP and BFRP confinement were both effective. For BFRP wrapping, the maximum axial strain was 2.1–3.2%, and its value for PAN FRP was 3.3–5.6%. At higher axial or lateral strain, the PEN FRP-wrapped concrete cylinders increase in strength as compared to PEN FRP wrappings. For the stress–strain relationship of confined concrete, a simplified rational modeling procedure is suggested.

In a study investigating the active confinement of concrete columns (shape-modified) and to check the effectiveness of passively confined analogous columns and actively confined shape-modified columns, monotonic compression tests were conducted [37], the HAP of shape memory alloy spirals were used for active confinement, and reused SMA wires (without HAP), CFRP, and BFRP were used for passive confinement. The results demonstrated that actively confined shape-modified columns significantly exhibited higher residual strength and ductility at larger axial deformation. Actively confined specimens showed low damage at failure. A total of 26% strength was increased when the confinement ratio was 0.11% for the passively confined BFRP mesh. It is suggested that an excellent alternative for the CFRP is BFRP for the unconfined concrete because it is lower in cost, as well as having good potential for strength and ductility increment. To evaluate the compressive behavior of FRP-confined steel-reinforced concrete (FCSRC) columns with high-strength concrete (HSC), an extensive experimental study was conducted by considering the thickness of the FRP tube and steel section shape [38]. The compressive behavior of FSCRC specimens under normal strength concrete (NSC), steel-reinforced concrete (SRC) specimens, and concrete-filled FRP tubes (CFFTs) was also investigated to make a brief comparison. Higher axial compressive capacity and higher axial stiffness for FCSRC columns were produced using HSC, as indicated by the study, even though the deformation capacity was reduced when compared with NSC use. In the GFRP tube, the strain distribution was more uniform with the GFRP tube in steel sections. To strengthen the existing SRC specimens with the use of GFRP tubes, EFS factors were proposed because, when HSC was used, the EFS factors were smaller.

To prevent the chemical attacks and segregation of concrete after confinement, the unplasticized polyvinyl chloride (uPVC) tubes were experimentally tested on concrete cylinders, both unconfined and confined, using uPVC [39]. Those cylinders were prepared using four types of uPVC tube sizes and five various concrete classes, with an aspect ratio of h/D = 2, and were ultimately tested under axial compressive load. As compared to the unconfined levels, the uPVC confinement increased the strength, energy absorption, and ductility factor between 1.28 and 2.35, 11 and 243, and 1.84 and 15.3 times, respectively. There was an inverse relation between 2t/D and the absolute value of slope in the stress–strain curve for post-peak behavior. There were several advantages seen when uPVC tubes were used: they protected the concrete from chemical attacks, acted as the permanent framework, prevented the segregation of concrete, were taken as a concrete cover and prevented peeling, and decreased the cross sections, which resulted in lighter sections. It was concluded that uPVC tubes could be used as confining material for electric poles, bridge piers, piles and highway sign boards. As the temperature went higher than 70 °C, uPVC decreased its strength, so it is suggested that uPVC confinement incorporate in the construction industry with an innovative fire protection mechanism. The geopolymers are also alternative solutions; in an experimental study, the properties of geopolymers were enhanced with the addition of banana fibers (BF) and fly ash (FA), which was used to make banana geotextile-reinforced geopolymer mortar (BGT-RGM) [20]. Thermogravimetric analysis (TGA), workability, scanning of electron microscopy (SEM) imaging and compressive and dog-bone tensile strength were used to evaluate BFRGMs. The concrete unconfined and confined with BGT-RGM was evaluated to develop stress–strain curves. The results showed that BGT-RGM, with a thickness of 20 mm and 15 mm and 20 mm geotextile grid spacing, indicated an increase in strength by 33.3% and 33.1%, respectively. It was suggested the life-cycle impact of the process and product, which could consist of the scalability of BFRGM making, be checked. It is also recommended that BGT-RGM technology’s potential application be explored in other fields like retrofitting arches, eaves, lintels, and masonry walls. Table 1 compares the different materials for concrete wrapping based on different properties and achieved performances.

## 3. Partial Confinement Methods of FRP for Enhancing the Strength of Concrete Columns

**Figure 2:**
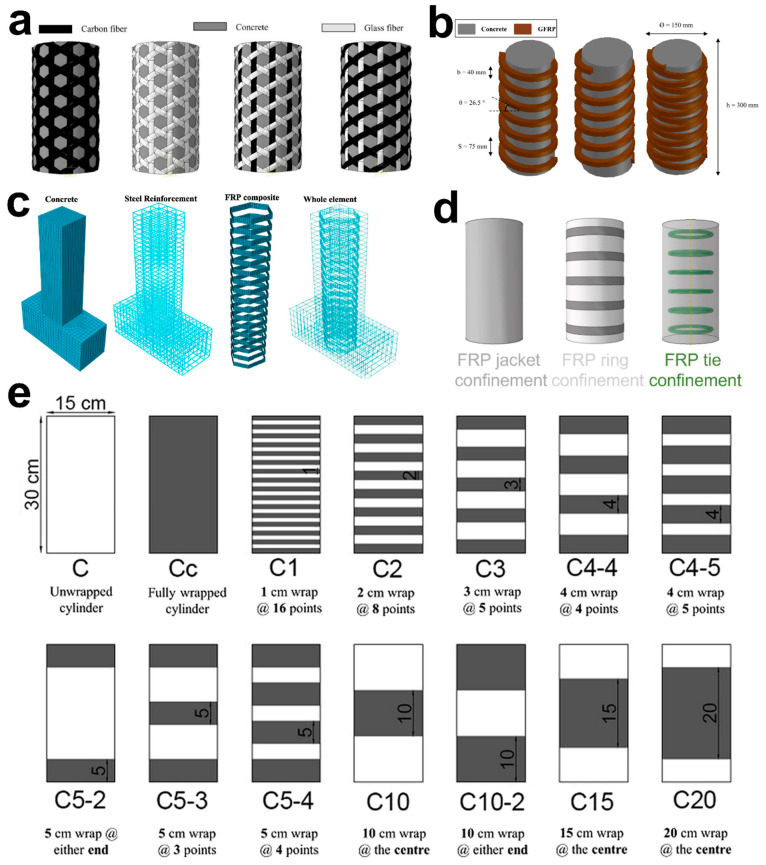
Different confinement configurations of concrete columns using different FRP composites. (**a**) Triaxial woven fabric wrappings with carbon and glass and hybrid configurations [19]. (**b**) Double spiral strips of GFRP for deteriorated concrete columns [46]. (**c**) FEM mesh for RC square columns and GFRP composite wraps [43]. (**d**) Different confinement systems of discontinuous FRP composite rings, internal tie confinement, and jacket confinement [47]. (**e**) Various patterns of CFRP wrappings on concrete columns using longitudinally laminates of discrete strips and partially wrapped at different positions and spacings [48]. Source: (**a**) is from an open-access article distributed under the Creative Commons Attribution License. (**b**–**e**) reproduced with permission [43,46,47,48] copyright © 2024, Emerald publishing, Elsevier, Springer.

**Figure 3:**
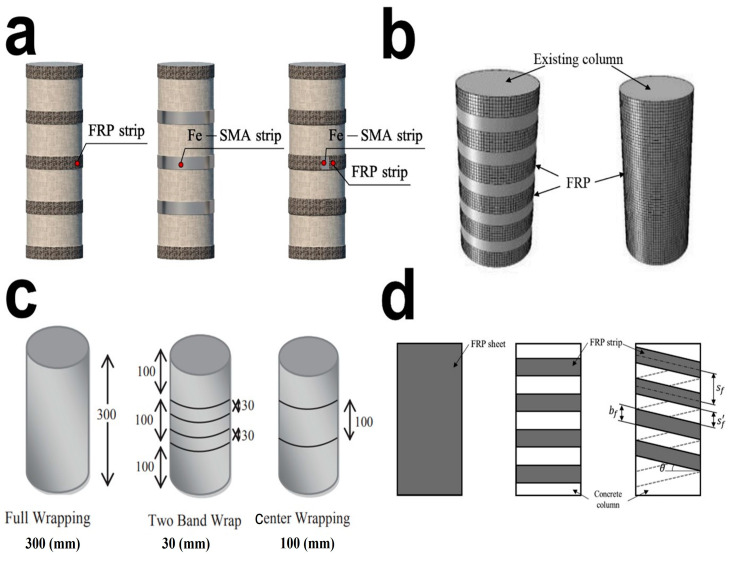
Novel configurations for partial confinement of concrete columns. (**a**) The different conditions of confinement are demonstrated with different spacing ratios and layers, where SMA is shape memory alloy [57]. (**b**) Different strengthening techniques are demonstrated as partial and full [58]. (**c**) Different wrapping patterns are studied for compressive strength (unit is mm) [59]. (**d**) The different arrangements for external FRP wraps using hoop and spiral strips for RC columns [60]. Source: (**a**–**d**) reproduced with permission [57,58,59,60] Copyright © 2024, Elsevier.

To address the deterioration or increased life span of concrete columns, the FRP stands as an effective confinement approach. It is inevitable to confine the specific regions of the columns to maintain the optimal material efficiency. Therefore, a few of advanced and novels studies for the effective partial confinement configurations of concrete columns by FRP composites are discussed here (Figure 2 and Figure 3).

A study aimed to evaluate two methods, i.e., horizontal and helicoidal strip confinements, for the CFRP partial confinement for specimens; then, a comparison was conducted for the partial and fully confined specimens [40]. The 20 mm strip spacing of partial horizontal CFRP strip confinement showed a load capacity of 80.56 MPa, with 71% strength enhancement. To strengthen concrete columns, partial CFPR-confined specimens with horizontal strips were equally sufficient as the fully CFPR-confined specimens. A study must be carried out to propose a unified stress–strain model for fully and partially confined FRP-wrapped concrete. Therefore, in a study, a simplified design equation was formulated by encasing three partially concrete columns and nine confined partially CFRP-encased concrete columns, which were axially tested. Load versus displacement relationship, failure modes, initial stiffness, ductility and axial bearing capacity were presented and analyzed [41] The outcomes showed that the mechanical performance of CFRP-confined partially encased concrete columns was better than that of those without CFRP sheets confining. It was recommended that with the increase in the number of CFPR sheet layers, the axial bearing capacity and ductility of columns could be improved significantly. For CFPR-confined partially encased concrete columns, a simplified design formula was proposed that could agree with test results satisfactorily.

The short concrete cylinders under axial compressive load were wrapped partially with GFRP composites [42]. The behavior of fully and partially confined cylinders was simulated using finite element software. The parameter was ‘x/h’, which was the ratio of the height of the confined zone to a total height of the cylinder. The results show that as the x/h ratio increased, there was an increase in ductility and ultimate strength, which directly affects the confined fc–εc relationship. The parameter of x/h could modify the behavior and improve the strength and ductility of confined concrete, as was suggested in the study. Good concordance was demonstrated as the experimental and numerical results were confronted. To further explore the finite element modeling (FEM) of concrete columns with GFRP strips designed in a hexagonal pattern, a simulation using ABAQUS under axial loading was developed [43]. Secondly, FEM was developed to understand a new reinforcement method for RC columns, which were partially confined under a monotonic horizontal loading. The results that were obtained clearly demonstrated that the columns’ performance was significantly enhanced with the designed GFRP strips. As the GFRP layers increased, the bearing capacity for load also increased by the values of 15–28.5% and 19–50%. For partially GFRP-confined large columns, the author recommended future design optimization and simulations to be conducted for more real physical aspects. It includes the interfaces of all consecutive materials and effects of columns components and partially confined concrete having hexagonal FRP strips, loading patterns like horizontal, axial and cyclic loading.

An experiment with varying FRP strip gaps was executed to make inquiries about the performance of confined concrete with partial FRP wrappings [44]. Axial stress, axial strain, axial load, and axial deformation behaviors were investigated for wrapped and no-wrapped concrete. Denison 5000 KN testing machine was used for the compression test. When the diameter of specimens exceeds the strip gap, then confinement effectiveness is negligible. The axial load axial deformation behavior was classified into four types for partially FRP-confined concrete based on the FRP strip gaps. The recommended confinement effectiveness coefficient was (1 − *s*/*d*^2^). A stress–strain model (K_e_) based on the Lam and Tang model was developed for partially FRP-confined concrete. The predictions of the developed stress–strain model was better than the existing models. To further generalize the spacing, 28 concrete columns wrapped partially with FRP and spiral strips were used to conduct axial compression tests [45]. The clear spacing width, thickness, and width of FRP strips and spiral angles were the test variables in this study. The effectiveness factor of the proposed confinement affects the ultimate axial stress and post-peak linear slope branch slope, the branch of the axial stress–strain curve. By varying the above-mentioned key parameters (the width, layers, and spiral angles of FRP strips), the desired confinement effectiveness factor could be achieved for the specimens. The recommended clear spacing ratio was s’_f_/D < 0.2, and the clear spacing to thickness of strip ratio was s’_f_/t_f_ < 100. The proposed model provided a precise prediction of the suitable design of FRP spiral strip-confined concrete.

Research using an alternative approach to the fully wrapped strengthening technique was conducted. Here, discrete FRP strips (rings) were used to wrap over circular concrete columns to investigate the axial compressive behavior of specimens [11]. Clear spacing and width of FRP were examined as the main test variables in that experimental program. Among the five representative models, one model proved to be superior to the other models. To attain monotonic ascending stress–strain behavior, the suggested s’_f_/d value must be higher than 0.5. However, if the predicted ultimate strain and stress were not underestimated at the turning points, the recommended model would perform best. For further stress–strain analysis, a study was conducted to determine the comprehensive difference between the mechanisms of partially and fully FRP-confined circular columns with normal-strength concrete [49]. Regression analysis was used to propose a formula that was used to determine the strength and corresponding strain on stress–strain curves. The results indicated that for the prediction of the ultimate conditions of partially FRP-confined concrete, the new model performance was better than the selected models. The strength of the ultimate point and peak point was related to actual confinement ratio (*f*’1, a/*f*’co), and the strain was the function of strain ratio ρ_ε_ and confinement stiffness ratio ρ’k. To distinguish between the stress–strain relationships of various specimens, it was suggested that the relationship between the actual confinement ratio and 0.07 be adopted as a criterion for the prediction of axial stress–strain curves; satisfactory accuracy and good agreements could be achieved using the new model.

An experimental study by [50] on 28 concrete columns was conducted to check various wrapping schemes. It was based on an arrangement method in which, from the middle zone to the end, the applied FRP material decreases. That was a kind of novel wrapping scheme proposed by research. The results suggested that along the height of the specimen, the FRP materials could be effectively utilized by using the proposed wrapping scheme. Existing wrapping schemes (full, partial, helical, and non-uniform) were compared to the recommended scheme with the same amount of FRP materials. That comparison indicated that strength, load-carrying capacity, the ductility of columns, and the utilization efficiency of FRP materials was improved significantly using the recommended scheme. It was suggested that combined loads (torsion, bending, and shear force) could be resisted with steel tube columns or reinforced steels along with the recommended FRP wrapping scheme. Another novel wrapping scheme was introduced, buckling restrained braces (BRBs), under a novel restraining system consisting of concrete panels that were confined partially to CFRP wraps to reduce the weight of the brace [51]. Initially, the verification of finite element analysis was performed; then, it was used to find the optimum positioning of CFRP wraps. A cyclic test was also performed on BRB frames. The brace endured until 2.5% drift, and after the end of 3%, they failed. That proposed system reduces the weight to 30% and also absorbs energy very well. The requirements of AISC-341 were satisfied by the proposed brace. To enhance the behavior of braces, the confinement at the end of restraining parts was recommended. To prevent any out-of-plane deformation, further supplement addition is recommended at the vulnerable connection parts that have less confinement.

An experimental study was conducted on 17 reinforced concrete columns to investigate the ductility and strength increment under the partial and full confinement of CFRP [52]. To study the jacket arrangement, the volumetric ratio of CFRP was kept constant. Then, the comparison of experimental load values was conducted between the theoretical value obtained from ACI and the fib guidelines and the experimental values. Although the results concluded that in all partially and fully confined columns, there was a considerable increment in strength and ductility, it varied with the location of the jacket. For partially wrapped columns, the strength increment was 59% for the 75 mm wrapping of two, and for 50 mm wrapping with of three, the increment was 83%. It was suggested that the designer could choose between ACI and fib for load-carrying capacity depending on the risk in the project, while the fib calculation, with a reasonably designed factor of safety (>2.0), could be used for both types of partial confinement. To further compare the full and partial confinement, 33 circularized square columns were molded and studied under axial compressive load. The specimen’s parameters included FRP volumetric ratio and FRP wrapping scheme, which consists of partially or fully wrapped, unconfined concrete strength and sectional shapes [53]. The test results indicated that the FRP confinement effectiveness and strengthening of square columns by the use of section circularized with the combination of FRP confinement could be improved by the section circularized of square columns, and it was also an economical and promising alternative to the strengthening technique of full FRP wrapping. According to this case, a model that could provide meticulous predictions for both ultimate axial strain and ultimate axial stress for partially FRP-confined concrete was suggested. For square columns, the effectiveness of FRP partial confinement could be enhanced by rounding the corners of square columns. Another study improved the effectiveness of FRP confinement by rounding the sharp edges of non-circular columns [54]. Therefore, the proposed circularization method achieved higher strength using partial wrapping.

To evaluate the effectiveness of partially wrapped strips, a stress–strain model was developed for the compressive stress–strain model (*f_c_-ε_c_*) of confined concrete [55]. Different parameters influence RC circular columns wrapped partially or fully with FRP, which were loaded concentrically, and a three-dimensional finite elements (FE) model was generated. The results indicated that with an increase in the number of FRP wraps, the ductility also increased, which unveiled that the confined *f_c_-ε_c_* got minor influence from longitudinal steel. Confined concrete compressive strength (f’_cc_) increased with the increment in unconfined compressive strength (f’_c_). The effect of transverse steel confinement (ρ_st_) decreased as the number of similar wraps increased (ρ_f_). A new model was proposed on a parametric study basis for partially and fully wrapped columns, focusing on axial strain and confined concrete compressive stress. The proposed model could predict the ultimate stress; however, other models overestimated its value. Another proposed FE approach was efficient in the prediction of ultimate axial strain and also compressive strength, exhibiting conservative axial behavior. In an experiment on 15 concrete column specimens under multi-axial compression, an accurate plastic damage model was employed [56]. The numerical results of the verified FR approach led to an improved understanding of concrete that is confined in partial FRP-confined concrete columns. Radial and axial stresses were more on the mid-plane of every FRP strip than on the mid-plane level of two adjacent FRP strips. For that reason, it was suggested that with the increase in concrete expansion, the confining stress became more non-uniformly distributed.

An experimental study was conducted on 40 concrete columns in seawater and sea sand, partially confined with CFRP, to analyze their axial compressive behavior [61]. Concrete type, number of CFRP layers, and CFRP strengthening scheme were the main variables of the test. The test results proclaimed that both CFPR-confined SCC and unconfined specimens depicted similar mechanical properties when compared to the respective specimens cast with normal concrete. For dilation properties and axial compressive behavior accountability, the two most important factors were the confinement stiffness of CFPR and clear spacing ratio. A new proposed model and several existing models were used and discussed to predict CFRP partially wrapped columns’ ultimate conditions. It was suggested that for the prediction of the ultimate conditions of concrete columns partially wrapped with FRP, more work had to be done both theoretically and experimentally. A new confinement model was proposed to predict the axial strain behavior of confined concrete columns. For concrete matrix, steel grid wires and partial hexagonal FRP were discontinuously embedded as strips [62]. In this work, under axial compression loading, a sum of 12 columns was casted and tested, including concrete-enhanced hexagonal FRP strip columns, concrete-encased double FRP grid strips columns, unconfined concrete cylinders, and concrete-encased steel grid strips columns. In the behavior of composite columns, there was a significant enhancement when using double concrete-encased strip columns. For partially confined concrete columns, the strength was improved by 30%, and the corresponding strain was increased by 140%. It is suggested that by decreasing the spacing between hexagonal GFRP strips and by increasing the number and amount of GFRP layers, the ultimate stress and axial strain could be sustainably increased for confined columns. A study checked the efficiencies of various confinements but also provided an effective and quick technique to increase the strength of already existing structural specimens [57]. In an experimental study, 18 columns were casted with a height of 450 mm and a diameter of 150 mm to investigate the compressive behavior of those concrete specimens under various spacing of different strips, confinement types, and different numbers of FRP layers. Active confinement, passive confinement, and hybrid confinement were the confinement methods. The results indicated that at the beginning, the hybrid and active confinement imposed their effects, while passive confinement was activated during the ascending period in the axial stress–strain curves. The load-carrying capacity and relative deformation of stub specimens was improved using the three types of confinements, but the performance of hybrid confinement was superior. To estimate the peak compressive stress of those columns, a calculation for verification (fl,a = k·ρsma·fsma) was proposed, proving that the coupling of Fe-SMA strips (Figure 3a) with the confinement of FRP was feasible. This innovative technique, with the use of Fe-SMA (shape memory alloy) strips, could repair the damaged columns caused by earthquake impact. Table 2 compares the methods of partial confinement based on the designed materials and recommended performance. Various novel confinements have been studied for better efficiency, such as FRP spiral strips as external strengthening configurations instead of FRP hoop/ring strips. A study showed that spiral strips have better fire resistance as compared to hoop strips [60]. To better understand the confinement mechanism of FRP spiral strips, a study conducted several axial compression tests and concluded that axial stress increased by decreasing the spiral angle [45].

For effective configuration of FRP fibers, it is recommended to have a horizontal orientation for concrete columns open to uniaxial compression forces to avoid expansion [65]. Further, the ideal configuration, along with the recommended spacing ratio/thickness and optimal positioning, are presented in Table 2.

## 4. Predictive Modelling and Simulation for Partial Confinement of Concrete Columns by FRP Wrappings

**Figure 4:**
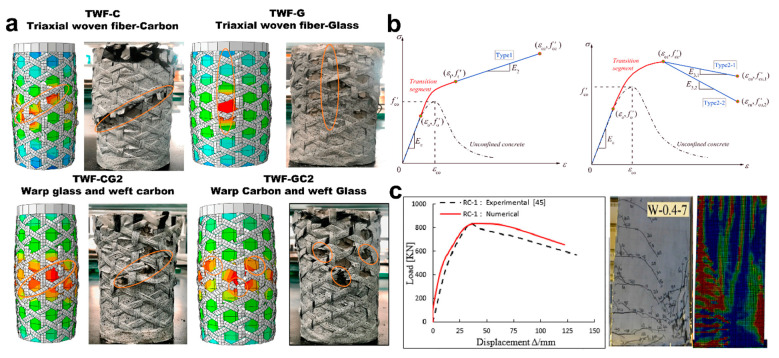
Simulation assessments and numerical predictions for different configurations of partial confinement using multiple hybrids. (**a**) The experimental results of four specimens reaching an ultimate capacity of axial loading resulted in the rupture of triaxial woven fibers of carbon and glass and hybrid configurations of glass and carbon, with a sudden decline in axial capacity. The figures on the right side are the pictorial proof of ruptures; however, the left pictures indicated FE simulations’ corresponding failure modes as shear and crush failure [19]. (**b**) The stress–strain curves showing three segments, including an obvious transition portion as a link connecting both linear segments, for partially confined circular concrete with FRP, where Type-1 is indicated as an ascending stress–strain branch, and Type-2 shows a descending post-peak response where type-2 is further classified in two branches for which the readers are suggested to this study [49]. (**c**) The load-displacement curves and failure modes of experimental and numerical results, validating proposed FEMs [43]. Source: (**a**) is from an open-access article distributed under the Creative Commons Attribution License. (**b**,**c**) reproduced with permission [43,49] Copyright © 2024, Elsevier.

It is now well established from the previous discussion and mentioned studies that partial confinement of FRP strips is an economical way and significantly enhances the strength of concrete columns. However, to make predictions of stress–strain in the ultimate conditions using precise confinement models and to make a thorough understanding of the currently developed models for partial confinement, a few studies are presented here (Figure 4).

A 3D FE modeling approach was developed based on a precise plastic damage model CDPM, and the concrete columns were compressed in a multi-axial manner [56]. The FRP partial wrapping technique was applied to fifteen column specimens. Comparisons were made between the numerical and test results for the validation of the FE approach. The arching action was verified by the numerical test results of confining and axial pressure distributions. The results showed that most ruptures occurred at the mid-point and initialized with the concrete cracking between adjacent strips. The stress–strain curves appeared in a bilinear shape, with peak stress showing a direct correlation with the width of FRP strips, whereas the ultimate strain was independent of strip spacing. However, the hoop and axial strains were both influenced by FRP strip spacing and layers. The loading function F was defined based on a specific mathematical expression, incorporating various parameters such as material strength and stress. The CDPM used a hyperbolic function to define the flow potential in the model. A one-fourth model was presented as a whole representation of the entire column due to the symmetrical shape. Concrete and FRP were modeled using elements of different types and correlated using ABAQUS. The results showed that the hoop strain distributions and the axial strain trend were closely aligned by FE and experimental observations. This limited set of test specimens might not be able to produce a generalized model; therefore, a new stress–strain model was developed for partially confined FRP concrete columns based on a new coefficient for effective partial confinement [44]. The strain hardening and softening responses of partially confined concrete columns were mainly considered under compressive tests with varying wrapping gaps. For the assessment of the proposed model, a large database of FRP partially confined concrete columns of 76 specimens was compiled, with the majority of them showing strain-hardening behavior. The database contained different properties of a specimen, such as diameter, unconfined concrete strength, and different FRP materials. In summary, the proposed model outperforms other models in the prediction of ultimate strength and strain and emphasizes the importance of FRP partially confined concrete columns.

The effect of the different arrangements of the wraps of carbon fiber-reinforced polymer (CFRP) was also studied on the confined concrete cylinders, and the numerical modeling for the axial stress–strain was introduced. The 3D non-linear FE model was verified using a series of axial-compression tests performed on differently proportioned laminated concrete columns by CFRP. Under positional changes and different widths of the partial wrapping of CFRP, the concrete columns showed a significant change in axial stress–strain. For FEM verification, the two specimens were considered as C and C10 based on axial stress–strain behavior. The numerical predictions and experimental results showed a good correlation in the stress–strain behavior of the C-specimen. The results of compressive strength comparison between the numerical and experimental results of different specimens showed that the compressive strength of the FE model was comparable to that of the experiments. The mean numerical-to-experimental compressive strength ratio was calculated as 1.03, hence proving that FE is a reliable model for measuring the confinement compressive strength of CFRP-wrapped specimens [48]. Detailed observations are required to be carried out further due to the typical failure pattern of discontinuously wrapped cylinders. By considering the possibility of concrete expansion at the vertical and horizontal directions in a non-uniform manner, a recent study introduced a new and unified analytical model for partially (FP) and fully (FF) FRP-confined concrete columns of square and circular cross-sections (SC and CC) when axial compression was applied and demonstrated an excellent performance of prediction [66]. The unification of key parameters for both fully and partially FRP-confined circular and square columns of concrete when subjected to axial compressive loading provided a more diverse evaluation criterion of the mathematical framework. When calculating the confinement pressure, the non-homogenous expansion distribution of concrete columns in both vertical and horizontal directions was also taken into account. In short, the active confinement approach determines the axial stress–strain curve by deriving the confinement pressure based on the dilation model. Through experimentation, the relationship of global axial stress–strain was also predicted in relation to FRP-confined column of concrete based on confinement stress path. Therefore, based on the variable pressure path of the confinement effect, the new axial strength model is based on the dilation method and comprises parameters from the global axial stress–strain curve of test specimens.

To incorporate the arching mechanism in partial confinement, a study presented an innovative design-oriented axial stress–strain model for the prediction of stress–strain relationships. Through regression analysis, the correlated stress–strain from the curves was also explored using formulas. To verify the model prediction, the data of 100 partially confined concrete columns were taken from the literature. As per the results, the model successfully captured the responses of the stress–strain characteristics. The main characteristics of the proposed model, such as dual stress–strain behaviors and three segmented curves, were taken from the stress–strain relationship proposed previously. It can be seen that the model incorporated two behaviors, strain hardening and softening, and two linear segments linked by a transition branch (Figure 4b). Before making predictions, the type of stress–strain behaviors is required to be known on the basis of material and geometric parameters. According to the results, for the first behavior, the proposed model precisely predicted the stress–strain responses for a normal stress concrete under partial confinement [49]. However, future work is required to establish more precise equations to estimate the strength and strain for the axial strain cases. To simulate the dilation and axial effects having analysis orientation, a model was recently introduced for partially confined reinforced concrete (RC) circular columns based on FRP and steel confinement [16]. The generalization of the expansion distribution of concrete in a non-homogenous manner increased the model prediction performance. The proposed model successfully predicted the dilation of RC circular columns and the effect of partial confinement. A new function for surface failure estimation was also introduced to simulate the vertical arching phenomenon and the expansion distribution of concrete along the column height. The model prediction results were validated by the experimental test results. For further comparison, the models by Teng and the concept of confinement efficiency factor were also considered. In order to develop new models for new confinement methods with new parameters of spacing and width and for new materials of confinement and concrete, the current model can be extended by recalibrating the failure surface function.

The inclined orientation of wrapping might be a solution for the varied failure patterns. Therefore, a predictive model was recently developed for the ultimate strain and peak stress when the FRP confinements were made at inclined orientations [67]. The confinement pressure was modeled as a function of fiber orientation angle based on non-linear regression methods. The two main approaches, analytical and numerical, were discussed for composite material mechanics and for design-oriented models. The inclination fiber cases were gathered to compile a database from 70 experimental results, in addition to some non-inclined fibers, for a more generalized approach. The dataset was split into a 70–30 ratio for non-linear regression training and later for the validation of the modified factors based on multi-expression programming (MEP). A precise correlation was generated among fiber orientations and confinement pressure by the modification factors ranging between 0 and 1. For fiber angles ranging between 3 and 45 degrees, the modifications were particularly effective for improved prediction accuracy. Further, to study the non-uniformity of partially confined concrete columns along the longitudinal direction, a 3D FEM, along with theoretical analysis exploring axial stress distribution ring confinement, was developed, focusing on unconfined areas between adjacent rings [47]. The FEM incorporated a better constitutive model (CDPM), and the optimal mesh size was estimated from previous studies. The axial stress–strain curves predicted by FEM aligned well with the experimental test results, but only a minor discrepancy occurred at the transition point between the first and second segments. The theoretical model was proposed for the angle of arching action, which comprised ring thickness, width, spacing, and unconfined concrete strength in correlation with the arching action angle. The new coefficient for effective confinement resulted in precise ring confinement in circular columns due to more parameters than the existing empirical coefficient and was defined as the ratio of decreased volume among two FRP rings to the total volume between them. According to the numerical results, the columns having the same confinement coefficient displayed slightly different stress–strain responses. However, the FE model predictions and theoretical model results were closely aligned for the axial distribution for the unconfined area between two adjacent FRP rings.

For the design optimization of GFRP-based partial confinement configurations, a non-linear finite element approach (NLFEA) was introduced to repair concrete columns subjected to axial compressive loadings [68]. Various confinement rates for FRP wrappings were assessed based on the non-linear behavior of strain and compressive strain properties input in ABAQUS-embedded numerical models (CDPMs). The Tsai-Wu failure method was incorporated into an orthotropic-elastic model for the prediction of FRP behaviors. The predicted numerical results of the proposed approach for different configurations of FRP strips, along with crack evolutions, were validated by the previous experimental models. The results showed that the proposed configuration significantly enhanced the mechanical performance for the repair work of concrete columns. The most critical parameters of FRP strips for the ultimate strength of confined concrete are estimated as the number of strips, width, and spacing. A 17.84% increment was observed in the compressive strength of confined concrete configuration (5CS1) compared to unconfined concrete. For instance, an ultimate strength of 38.14 MPa was achieved by the five strips of 40 mm width. Further experiments and simulations, along with analytical models, are important for more comprehensive solutions. To incorporate the wrapping effects in the reinforced columns having discontinuous steel grips, a recent study by the same authors performed the FEM for hexagonal glass fiber-reinforced polymer (GFRP) strips under partial confinement of RC and concrete columns when applied both axial and horizontal loadings [43]. The stress–strain responses under axial loadings were presented by the simulation results (Figure 4c). A new FEM was also established for an innovative reinforcement method for the partial confinement of RC columns at a larger scale. Therefore, the new model is based upon monotonic loadings in the horizontal direction for the new partially reinforced FRP design of the real RC columns. The 3D numerical simulations incorporated the non-linear behaviors of the given materials. For validation of the numerical results of FEM, comparisons were made with the previous experimental results in terms of failure mechanisms and carrying capacity curves. Further, it was recorded that the hexagonal FRP strips on discontinuous steel grips incorporated by concrete matrix enhanced the ductility and lateral carrying capacity to 67 and 16%. When the FRC-1 and FRC-2 configurations were applied, the capacity was significantly increased by 15–28.5% and 19–50%. The predicted results mainly concluded that the enhanced strip thickness and reduced spacing greatly increased the strength of RC columns. However, various loading conditions, embedding strip patterns, and material properties are required to be investigated in future simulations.

Machine learning (ML) models solve problems in almost every field of life due to their ability to learn complex problems and make precise predictions. The torsion strength was predicted for the external confinement of FRP on RC columns using a neural network [69]. The training dataset was collected from previous experimental studies. The input parameters were effective depth, reinforcement ratio, fiber orientations, and different schemes of strengthening. The model achieved prediction accuracy with a 0.93 coefficient of determination and a mean absolute error of 0.98. The results further showed that NN surpasses the other models, such as ensemble tree and Gaussian regression; however, the brittleness of torsion-FRP behavior could be predicted by the complex scenarios. The large-scale databases of multiple confinement configurations, performance parameters, and material ratios are necessary for efficient models. To further explore the ML models, a recent study applied genetic expression programming (GEP) on CFRP-confined columns for compressive strength prediction [70]. An extensive database of 828 datasets was compiled, and different models were trained to evaluate the best one. The GEP performance was better than NN in predicting the compressive strength of circular columns of concrete. More ML models are required to be trained on the specific cases of partial confinement of concrete. ML offered some challenges along with precision, as for better results, extensive training data covering all aspects and factors is required. However, the lack of a theoretical basis is a big setback in justifying the predictions.

A thorough comparison between different modeling techniques for the partial wrappings of concrete columns, along with key parameters, is presented in Table 3. It is now well-established that the FEA and analytical models are inevitable for the analysis of wrapping materials, configurations, concrete structures, and confinement methods. However, a brief comparison is an elaboration between the pros and cons of both. The FEA is a better option for generalization and complex designs as compared to analytical models. However, the analytical models require fewer computational resources and have a better theoretical basis as compared to FEA.

## 5. Comparative Analysis and Correlation between Confinement Materials, Methods of Partial Confinement, and Predictive Modeling and Simulation for Effective Partial Confinement of Concrete Columns

It is essential to research the integration and optimization of the materials, methods, and predictive modeling. It is equally important to consider the environmental conditions, structural design, and cost for better optimization. For confinement materials, the different types are glass, carbon, jute, organic, and hybrid fibers, which have different mechanical properties and characteristics. The different properties mandatory for effective confinement are strength, strain, and stiffness. These are equally important along with the confinement methods such as partial spiral, hoop, etc., for specific designs.

In lieu of the above discussion, the following relationships are established for the effective partial confinement of concrete columns. The combined effect of material and application method significantly influences the performance. For instance, the high-performance composite would provide the design strength if wrapped in an effective way that is tailored to the specific structure and deteriorating part. However, predictive modeling helps optimize materials and configurations for practical and durable partial confinement solutions. Therefore, the model and simulations must be updated for the new materials and advanced configurations.

### 5.1. Overall Challenges and Future Perspective

The emerging designs and huge structures are bringing new challenges for researchers in the fields of material sciences, textile engineering, structural engineering, environmental sciences, urban planning, etc. It is equally important to mention these challenges for the upcoming researchers and suggest possible solutions for them.

### 5.2. Challenges

Developing lightweight and high-performance composites with repeated quality under various loadings enhancing compressive strength, ductility, and durability, while reducing cost remains a significant challenge. In addition, the resistance to chemical attacks is required to be enhanced for confinement to concrete columns in adverse conditions.

1. There are very few studies on the FRP-concrete bonding behaviors, however, an adequate bonding strength is imperative to ensure the uniform stress transmission between the FRP and concrete columns.
2. The research gap in various confinement methods for partial confinement is limiting the selection of most suitable method for long-term applications and bending analysis over a long period. For instance, the certain confinement limits for FRP hoops and spiral strips are unknown to avoid concrete softening.
3. There is less data on the fiber orientations and confinement angles for improved ductility under seismic cycle loadings.
4. Less work is available on the external environmental factors, and studies on the partial FRP-confined concrete columns are stressed.
5. The large-scale database of experimental results for partially FRP-confined concrete columns is not available freely.
6. For flexural members like beam-columns joints and bridge girders, the FRP partial confinement adds some challenges including, durability of bonding between surfaces of concrete and FRP material, stress concentration under repeated loadings, and load discontinuity, etc.

### 5.3. Future Perspective

The multiple process variables as compression pressure, temperature, and cooling rate, etc. of new composites manufacturing for partial confinement of concrete columns are required to be design specific and environment friendly.

7. Under different loading conditions, the bonding analysis, such as the bond-slip relationship of the concrete-FRP, is necessary for long-term performance. Dynamic loading conditions are essential to study bond-slip behavior for a better understanding of FRP-concrete systems. Cyclic loading, fatigue loading, and impact loading can be incorporated into the advanced simulation techniques by considering amplitude, frequency, number of cycles, stress range, mean stress, resistance, peak load, impact, and response to high stress–strain rates along with time-dependent loading conditions, etc.
8. It is important to perform both experimental and numerical studies on concrete columns partially confined with different configurations of FRP systems to investigate compressive and axial compressive stress–strain behaviors, crack evolution and failure morphology, long-term deflection behavior of flexural concrete structures, etc.
9. It is essential to study the different fiber orientations of composites for ideally aligning to the internal stress distribution of concrete structures, to enhance the mechanical properties and compressive strength. Horizontal and vertical directional fibers can enhance ductility under seismic loadings, helically wrapped fibers can increase lateral and axial strength. Therefore, various other different orientations should be explored for specific designs.
10. The simulations and predictive models are required to be extended to incorporate the environmental factors and external stresses for long terms stability of partially confined concrete columns. For further research in this domain, scientists must explore potential modeling techniques including fatigue and fracture mechanics models, Monte Carlo simulations, accelerated aging tests, and coupled multi-physics simulations, etc.
11. For effective predictive modeling and simulations, it is imperative to develop extensive databases for different configurations, and materials of concrete column confinement. It requires collaborative research projects between academia and industry to build such comprehensive databases. Open-access repositories with standardized protocols can help to promote data collaborations and reproducibility.
12. Developing hybrid composites of carbon, jute, glass, etc. can add properties of all materials including strength, stiffness, ductility, and toughness. Sustainable plant-based natural fibers can help reduce the environmental impacts if can provide high-performance mechanical properties.

### 5.4. Conclusions

Precise simulations and analytical models are inadequate to predict the material properties of fiber-reinforced polymers (FRP) and partial confinement configurations for enhancing the strength of concrete columns. Optimized partial confinement using materials with high tensile strength, durability, lightweight, and corrosion resistance would improve the ductility as compared to full wraps. The efficient models for partial confinement are an active area of research for understanding the mechanical properties of large-scale structures and life spans. This study spotlights the latest insights and synergy among the materials, confinement methods, and predictive modeling for effective partial confinement of concrete columns.

## Figures and Tables

**Table 1:** Comparison between strengthening techniques for concrete columns confinement.

Concrete Columns Material	Wrapping Material for Concrete	Properties of Wrapped Material	Performance	Reference
**Reinforced concrete**	**“Hybrid Composites”** carbon fiber fabric wrap, glass fiber fabric and hybrid fiber fabric wrap	CFRP modulus of elasticity = 238 GPa GFRP modulus of elasticity = 76 GPa CFRP tensile strength = 3650 MPa GFRP tensile strength = 2200 MPa Area density (CFRP) = 225 g/m^2^ Area density (GFRP) = 430 g/m^2^	Load bearing capacity increase = 22% Improvement in ductility = 1.65	[21]
**RC short columns**	**“Hybrid Composites”** CFRP wrap and steel collar strengthening	Collar Tensile Strength = 452 MPa CFRP Tensile Strength = 1900 MPa Modulus of Elasticity collar = 161,857 MPa Modulus of Elasticity CFRP = 230,000 MPa	Ductility increment for CFRP = 38–108% Ductility increment for collar = 61–100%	[34]
**BFRP & ECC** **Confined concrete**	**“Hybrid Composites”** Basalt fiber textile Textile reinforced ECC	Size: 25 × 25 mm weft and wrap formed by monofilaments Density: 120 g/m^2^ Tensile strength: 658.7 MPa	BFRP and ECC tensile strength = 3.7 MPa BFRP and ECC tensile strain = 0.00657	[35]
**Plain concrete**	**“Hybrid Composites”** Polyethylene naphtholate fiber-reinforced polymer (PEN FRP) and Basalt FRP (BFRP)	Density of BFRP fiber = 0.0021 g/mm^3^ Density of PEN FRP fiber = 0.0014 g/mm^3^ Tensile strength BFRP = 1226 MPa Tensile strength of PEN FRP = 842 MPa Elastic modulus of BFRP = 68.4 GPa Elastic modulus of PEN FRP = 17.5 GPa	Axial strain for BFRP = 2.1–3.2% Axial strain for PEN FRP = 3.3–5.6%	[36]
**SMA alloy** **Spirals** **and plain concrete**	**“Hybrid Composites”** CFRP and BFRP	BFRP density = 2.75 g/cm^3^ CFRP density = 1.76 g/cm^3^ Elastic modulus BFRP = 82 GPa Elastic modulus CFRP = 252 GPa Tensile strength BFRP = 1602 MPa Tensile strength CFRP = 4300 MPa	Strength of BFRP > Strength of CFRP	[37]
**Steel-reinforced HS concrete**	**“Hybrid Composites”** GFRP tubes	Compressive strength = 161 MPa Axial elastic modulus = 11.5 GPa Hoop Poisson’s ratio = 0.41	-	[38]
**Compositional** **Varied concrete of 4 types**	**“Hybrid Composites”** Un-plasticized polyvinyl chloride (uPVC) tubes	Ultimate tensile strength = 49.5 MPa Young’s modulus = 3.5 GPa Poisson ratio = 0.34	Strength increment = 1.28–2.35 times ductility factor = 1.84–15.3 times energy absorption increment = 11–243 times	[39]
**M15 concrete mixture**	**“Hybrid Composites”** Banana geotextile-reinforced geopolymer mortar (BGT-RGM)	Diameter of BFRGM = 13.98 cm Compressive strength = 24.57 MPa Thickness = 13 mm Tensile strength = 0.79 MPa	Tensile strength improvement = 83% Compressive strength improvement = 33%	[20]
**Steel-reinforced concrete columns**	E-glass fiber reinforced cementitious matrix (GFRCM)	Overall area weight = 251 g/m^2^ Elastic modulus of fiber = 64.4 GPa Ultimate tensile strength = 525 MPa Fiber ultimate tensile strain = 0.9% Equivalent thickness = 0.05 mm	Strength improved = 20–30%	[27]
**Concrete containing colored waste glass**	polypropylene textile	Thickness = 15 mm Width = 55 mm Tensile strength = 1265 MPa Young’s modulus = 8698 MPa	Resistance increases up to = 34.78 MPa	[28]
**Reinforced concrete**	CFRP type (M1, M2, M3)	Axial tensile strength = 4800 MPa Axial modulus of elasticity = 240 GPa Rupture strain(axial) = 2%	Axial strain = 1.9–2.2%	[23]
**Low-strength hybrid fiber-reinforced concrete**	Carbon fiber-reinforced polymer (CFRP)	Density of fiber = 1.65 g/mm^2^ Fiber thickness = 0.12 mm Tensile strength = 3500 MPa Modulus of elasticity = 28 GPa Ultimate strain = 1.67%	Increase in CS = 113.9% Axial compressive strength = 34 Mpa	[24]
**Hybrid fiber-reinforced polymer (HFRP) spiral confined concrete**	Basalt and carbon fiber	Density of Basalt fiber = 2.6 g/cm^3^ Density of Carbon fiber = 1.85 g/cm^3^ Tensile strength of Basalt fiber = 2250 MPa Tensile strength of Carbon fiber = 3000 MPa Elastic modulus of Basalt fiber = 90 GPa Elastic modulus of Carbon fiber = 210 Gpa	Ultimate strain of HFRP spiral > ultimate strain of CFRP bar	[25]

**Table 2:** Different confinement configurations of partial confinement of FRP on concrete columns.

FRP Material	Confinement Spacing/Thickness/Width	Strength of Unconfined vs. Partial Confined	Recommended Spacing Ratio/Thickness	Optimum Positioning	References
**Fe-SMA strips and BFRP strips**	Strips spacing = 30, 40, 75 mm Number of strips = 3, 5 and 7	Compressive strength of Fe-SMA with 3 strips increased = 71.10 MPa Compressive strength of FRP with 3 layers = 53 MPa	Narrow net spacing	Strip spacing = 30 mm Number of strip layers = 3	[57]
**Carbon fiber-reinforced polymer (CFRP)**	Thickness of CFRP layer = 0.25 mm	Strength of proposed confinement = 86–120 MPa	Stronger confinement in the middle with width = 100 cm	Middle zone wrapping with a decrease toward the end	[50]
**Fiber-reinforced polymer (FRP)**	Width of FRP strip = 50 mm	Compressive strength of fully wrapped = 20–30 MPa Compressive strength for partial = 55–70 MPa	Width of strip = 40 mm	increase in number of strips = 1–7	[55]
**CFRP**	Thickness of layer = 0.37 mm	Max stress in CFRP strip = 490 MPa	Based on a smaller s/L ratio.	M-5 and M-8 coverage area = 50% M-9 coverage area = 40%	[51]
**CFRP**	Width of strips variation range = 1 cm–20 cm	Compressive strength of unconfined = 36 MPa Compressive strength for partial confinement = 40	Spacing between the strips = 1–2 cm	Wider CFRP wrap in the center	[48]
**CFRP**	Thickness of layer = 0.167 mm	Axial stress of unconfined = 36.4 MPa Axial stress of partially confined = 70 MPa (average)	Spacing between strips = 25–35 cm	Less spacing between strips was recommended	[44]
**FRP**	Thickness of strips range = 0.167–0.334 mm Width of strips = 25, 30 and 35 mm	-	-	More FRP strip width with 4–5 FRP strip	[11]
**SikWrap-230 C unidirectional CFRP**	Thickness of strip = 0.131 mm Width of strip range = 100–600 mm	Elastic stiffness for unconfined = 222 MPa Elastic stiffness for partial confinement = 3000–7900 MPa	Width for the confinement = 300–400 mm	Strips with a confinement width of 500 mm for low-strength concrete	[63]
**FRP**	Width of FRP strip = 25, 30, 35 mm Thickness of strip = 0.167 mm	Compressive Strength for partial confinement = 23–27 MPa	-	Increase in the thickness of the strip increases the compressive strength	[56]
**CFRP**	Thickness of strip = 0.167 mm Number of CFRP layers = 0–3	Yield displacement = 11–13 mm	Lateral directional confinement of CFRP layer.	Axial bearing capacity of the column increased to 52% by 3 layers of CFRP	[41]
**Bidirectional fiberglass mat (GFRP)**	Hexagonal GFRP strips with width = 30 mm GGFRP strip thickness = 0.35 mm	Compressive strength of hexagonal GFRP = 32 MPa	Spacing between strips = 10 mm	Decrease in spacing of hexagonal strips Increase number and amount of GFRP layer	[62]
**SikaWrap-301C-CFRP**	Spacing of strips = 20, 40 and 60 mm	Compressive strength of horizontal strip Partial-CFRP = 80 MPa Compressive strength of helicoidal strip Partial-CFRP = 68 MPa	Spacing between the strips = 20 mm	partial CFRP confinement with a horizontal strip	[40]
**Bidirectional GFRP and a unidirectional CFRP**	Central zoning confinement with GFRP or CFRP	Compressive strength of partial CFRP = 41–45 MPa Compressive strength of partial GFRP = 40–42 MPa	Partial confinement in the central zone and Partial confinement with hybrid CFRP and GFRP	Two CFRP layers in the central zone or hybrid confinement	[64]
**GFRP**	Spacing between hexagonal strips = 30 mm FRP thickness = 0.35–1.4 mm	Compressive strength of unconfined column = 27.3 MPa Compressive strength of partially confined columns = 30–35 MPa	-	Increase of CFRP layers.	[43]
**CFRP**	Width of CFRP strips = 30, 40, 50, 60 mm Spacing between strips = 30, 60, 75, 90, 105 mm	Average compressive strength for partial confinement = 40 MPa	Width of CFRP strip = 45 mm Spacing between the strips = 30 mm	smaller clear spacing between two adjacent strips	[61]

**Table 3:** Predictive modeling for partial confinement of concrete columns.

Modeling Approach	Constitutive/Proposed Model	Confinement Method	Key Parameters	Validation Approach	Remarks	References
**FE modeling**	Concrete damage plastic model (CDPM), as proposed and improved by [71]	Continuous and discontinuous FRP strips	Effective confining pressure, yield function F, hardening function	Comparison of numerical test results with experiments	Few experimental specimens for modeling FE numerical results are not provided for partially FRP concrete columns	[56]
**Analytical model-an extension of existing stress–strain model**	An extension of the existing model with a new coefficient in the model [72]	Partially confined strips with varying gaps	Confinement effective coefficient K_e_, Strain hardening and softening, a gap of strip wrapping	Comparison with an experimental database of 76 partially FRP-confined concrete	Limitations in test conditions and complexity in strain distribution.	[44]
**FEM verification and comparison**	Theoretical stress–strain model by [73]	Different wrapping patterns of fibers	Axial stress–strain, confinement pattern thickness, position and ratio, compressive strength	Comparison between FEA, experimental and Youssef et al.	Similar damage patterns by fully and discontinuously wrapped cylinders	[48]
**Analytical model**	Basic framework of axial stress–strain by [74]. Active confinement approach–dilation model	Full and partial	Confinement efficiency factor and stiffness index	Regression analysis technique	The iterative process for R_1_ and R_2_ values is not clear	[66]
**A design-oriented axial stress–strain model**	Design-oriented model [11] Separate equations for type 1 and 2 models	Fully and partially	Actual confinement ratio, clear spacing ratio, hoop rupture strain of FRP	A large experimental database of FRP partially wrapped normal strength concrete columns	Emphasize the material properties, geometric parameters, and some specific ratios.	[49]
**Analysis oriented model**	Unified dilation model for axial deformations due to damage in unwrapped areas	FRP full and partial	Spacing, steel hoop/spiral, FRP confinement stiffness	Existing experimental results, previous model and confinement efficiency factor	Recalibration is required to adapt this model for new material and confinement types	[16]
**FEM-an innovative configuration of embedded strips**	3D simulations were tested on the basis of [75]	Hexagonal GFRP strips wrapped on discontinuous steel grid within the concrete column	Failure mode, lateral carrying load capacity, concrete strength, ratio	By experimental and analytical data for axial stress–strain and failure parameters	Simulation and data of large-scale RC columns needed for further optimization	[43]
**Existing stress–strain model with advanced FE approach**	Revised analysis model based on [76], Proposed arching action angle model, Improved CDPM by [71]	Concrete confined with FRP rings/ties	Confinement coefficient Kv,new = Ve/V_v_ Unconfined concrete strength, FRP width and thickness,	Comparing experimental evidence as stress–strain curves, failure mode, ultimate axial stress–strain distribution	Varied axial stress distribution in columns having the same confinement coefficient	[47]
**Analytical and numerical (Design oriented)**	Proposed factors based on the relations proposed by [15]	FRP inclined wrappings	Peak stress, ultimate strain, modification factors β1 and β2 as fiber angles	Experimental database 70% for modification and 30% for validation	Improvements for existing models but new models not presented	[67]
**Non-linear Finite element approach (NLFEA)**	CDPM, Tsai-Wu failure criteria	FRP stirrups	Strips spacing/width/thickness/rate of confinement	Comparison with numerical results of already available experimental data	Ensuring optimal adhesion in the bonded area is critical for repairment	[68]

## Data Availability

The data are already shared in the manuscript.
